# Sound attenuation in the ear of domestic chickens (*Gallus gallus domesticus*) as a result of beak opening

**DOI:** 10.1098/rsos.171286

**Published:** 2017-11-15

**Authors:** Pieter G. G. Muyshondt, Raf Claes, Peter Aerts, Joris J. J. Dirckx

**Affiliations:** 1 Laboratory of Biophysics and Biomedical Physics, University of Antwerp, Groenenborgerlaan 171, Antwerp 2020, Belgium; 2 Functional Morphology, University of Antwerp, Universiteitsplein 1, Antwerp 2610, Belgium; 3 Department of Mechanical Engineering, Free University of Brussels, Pleinlaan 2, Brussels 1050, Belgium; 4 Department of Movement and Sport Science, University of Ghent, Watersportlaan 2, Ghent 9000, Belgium

**Keywords:** avian ear, middle ear vibrations, laser Doppler vibrometry, bird vocalizations, audio recordings

## Abstract

Because the quadrate and the eardrum are connected, the hypothesis was tested that birds attenuate the transmission of sound through their ears by opening the bill, which potentially serves as an additional protective mechanism for self-generated vocalizations. In domestic chickens, it was examined if a difference exists between hens and roosters, given the difference in vocalization capacity between the sexes. To test the hypothesis, vibrations of the columellar footplate were measured *ex vivo* with laser Doppler vibrometry (LDV) for closed and maximally opened beak conditions, with sounds introduced at the ear canal. The average attenuation was 3.5 dB in roosters and only 0.5 dB in hens. To demonstrate the importance of a putative protective mechanism, audio recordings were performed of a crowing rooster. Sound pressures levels of 133.5 dB were recorded near the ears. The frequency content of the vocalizations was in accordance with the range of highest hearing sensitivity in chickens. The results indicate a small but significant difference in sound attenuation between hens and roosters. However, the amount of attenuation as measured in the experiments on both hens and roosters is small and will provide little effective protection in addition to other mechanisms such as stapedius muscle activity.

## 1. Introduction

As opposed to the middle ear (ME) of mammals that is built up of three ossicles, birds only have a single ossicle, the columella. Apart from this single bony ossicle, the avian ME contains a trifurcated cartilaginous extracolumella which joins the tympanic membrane to the columella and of which the central arm, the extrastapedius, gives the tympanic membrane its conical shape with the apex pointing outwards into the ear canal. The ear also contains a single muscle, the stapedius, that is innervated by a branch of the facial nerve and is located for the most part outside of the ME cavity [1]. Furthermore, the system is suspended by a series of ligaments, such as the annular ligament surrounding the columellar footplate, Platner's ligament that connects the columella to the quadratosquamosal articulation, ascendens ligament coupling the tympanic membrane to the extrastapedius and some drum-tubal ligaments that support the membrane [2].

**Figure 1.**
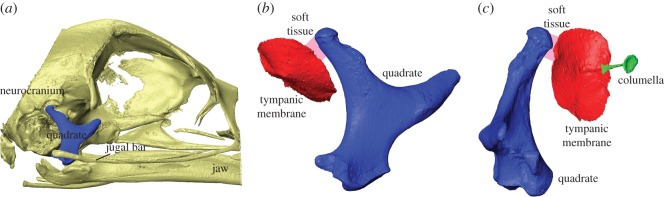
Geometry of the ear structures of the chicken's skull obtained from reconstructions of micro-CT images presented in Claes *et al*. [7]. (*a*) Lateral view of the chicken's skull illustrating the geometric relation of the quadrate (blue) to the neurocranium, jaw and jugal bar (yellow). (*b*) Enlarged lateral view and (*c*) frontal view of the quadrate (blue), tympanic membrane (red) and columella (green). The otic process of the quadrate attaches to the tympanic membrane via soft tissue (pink-shaded area). Beak opening goes along with rotations of the quadrate via the jugal bar, which leads to deformation of the tympanic membrane (relaxation or tension) by way of the soft tissue connection.

In contrast to the mammalian ME, which is embedded in a single bony cavity within the temporal bone, the avian ME and tympanic membrane are enclosed by two separate bony structures: the temporal bone of the neurocranium and the quadrate. The quadrate is a part of the beak suspension that can move relative to the neurocranium. Upper jaw and quadrate movements are linked via the pterygoid–palatine complex and the jugal bone (e.g. [3]). Additionally, the tympanic membrane is fixed at its rim by loose connective tissue in the ventrolateral part of the ME [4]. Furthermore, the cavity of the avian ME is connected to the contralateral ME via the pharyngotympanic tube and a complex-shaped intracranial air space (e.g. [5,6]). The geometry of the tympanic membrane and the columella of the chicken and their relation to the quadrate and the beak are shown in figure 1. The geometry is based on the reconstruction of micro-CT images that were presented in Claes *et al*. [7].

Sound transmission through the avian ear is influenced by the stapedius muscle, which was shown to react during self-generated vocalizations in the domestic chicken (*Gallus gallus domesticus*) based on electromyography measurements [8]. Grassi *et al*. [9] later claimed that this muscle activation mostly plays a role in vocal development, rather than effectively protecting the animal from its own generated vocalizations. Interestingly, Counter & Borg [8] concluded that mechanisms other than muscle activity must be present to account for the observed change in ME volume during vocalization, which influence ME function. Suggested explanations were muscle contractions in the outer ear, the insertion of air into the intracranial cavity via the pharyngotympanic tube or skull deformations during vocalization.

On top of these factors, additional mechanisms may play a role in the adaptation of ME function. Ewald [10] discovered that inner-ear pressure rises as a consequence of beak opening in pigeons. This happens due to the attachment of the tympanic membrane to the skin of the ear canal, which is connected to the skin covering the lower jaw: when the beak opens, the skin of the jaw is stretched, so it pulls on the tympanic membrane. The so-established static pressure is transmitted to the inner ear by the columella. Bray & Thurlow [11] investigated this hypothesis with cochlear potential measurements in pigeons, demonstrating an average drop of 20 dB in auditory sensitivity when the beak is widely opened.

As the tympanic membrane is connected to the kinetic quadrate and loose connective tissue, as illustrated in figure 1, it was proposed by Starck [4] that movements of the upper jaw, for instance during vocalization, may change the shape and tension of the tympanic membrane and thus affect hearing in birds. Claes *et al*. [7] compared the effect of maximal beak opening on the ME structures in the male and female domestic chicken using micro-CT, given the large difference in vocalization capacity between the sexes. It was found that tympanic membrane deformation, causing relaxation of the membrane, was more pronounced in roosters than in hens. These deformations may alter the acoustic properties of the tympanic membrane and could be part of a protective mechanism, possibly only present in roosters, which serves to prevent damage of the inner-ear receptor cells during vocalization, as in pigeons [11].

To investigate this hypothesis, the transmission of sound through the ear of the domestic chicken is investigated and compared for different beak-opening configurations by measuring vibrations of the ME. Up till now, only a few studies have reported measurements of ME vibrations in bird species. The few techniques that were used in this context include macrophotography on different animals [12], Mössbauer spectroscopy on doves [13] and pigeons [14,15], and capacitive probe to compare the tympanic membrane response in pigeon, parakeet, canary, cowbird and neonatal chick [16]. More recently, laser Doppler vibrometry (LDV) was used to measure tympanic membrane response in the neonatal chick [17]. LDV was also used to study the internal coupling of MEs in the framework of directional hearing in starlings [18], quails [19], budgerigars [20] and owls [21]. The same technique was used to study vibrations in the ME of the ostrich [22,23] and the duck [24]. In the present study, the vibration velocity of the footplate was measured with LDV *ex vivo* in response to sound pressures introduced at the entrance of the ear canal. These experiments are performed on both hens and roosters, and repeated for closed and maximally opened beak conditions. Because it is the aim of the present study to isolate the purely mechanical effect of beak opening, measurements are performed *ex vivo* so that other active mechanisms such as stapedius muscle action are excluded.

To demonstrate the need of a protective mechanism against potentially very loud self-generated vocalizations, audio recordings of a crowing rooster are presented. The acoustic properties of the rooster's crows are studied by analysing the amplitude and frequency content of the recordings. To verify their potential impact on the hearing of the animals, they are related to the results of the vibration experiments.

## 2. Material and methods

### 2.1 Middle ear vibration experiments

#### 2.1.1 Sample preparation

The heads of five male and five female young adult domestic chickens (*Gallus gallus domesticus*) between 3 and 13 months old were obtained from a poultry farm and were refrigerated at a temperature of 5°C for no more than 5 days before measurement. The subjects were euthanized in the function of the food industry, so no animals needed to be sacrificed for the current work. The adult males had a body weight of (2.4 ± 0.14) kg and females had a weight of (1.8 ± 0.12) kg. The domestic chicken was chosen because the species is widespread and well-studied, but also because of the large difference in vocalization capacity between the sexes. To measure ME vibrations at the medial side of the footplate, the skull was opened from the caudal side of the head using a band saw as shown in figure 1*a*, without damaging the hearing organs or any structures associated with the bill. To gain optical access to the footplate, the medial wall of the inner ear was opened from the inside of the skull and the remaining inner-ear fluid was removed from the surface of the footplate. Furthermore, the head was fixed in the experimental set-up by inserting a screw in the upper part of the skull. For the experiments with an opened beak, a plastic rod of appropriate length was placed between the upper jaw (i.e. the maxilla and premaxilla) and the lower jaw (or mandible) to open the beak maximally.

#### 2.1.2. Experimental set-up

To induce ME vibrations, a 10 cm diameter loudspeaker (Visation, FR10---4 Ohm, Haan, Germany) was used as an acoustic stimulation source. The sound generated by the speaker was concentrated by a funnel onto a small surface area of around 1 cm^2^, to concentrate the acoustic input at the entrance of the ear canal. To prevent significant sound pressure from reaching the medial and lateral surface of the columella, modelling clay was used to seal the sound source funnel to the ear canal. Stepwise pure-tone sinusoidal signals from 0.125 to 4 kHz, with 16 lines per octave, were delivered to the speaker via a custom-made amplifier. The frequency range was chosen in accordance with the hearing range of chickens [25]. A probe tube microphone (Brüel & Kjær, Probe Microphone Type 4182, Nærum, Denmark), connected to a conditioning amplifier (Brüel & Kjær, Nexus Type 2690-A-OF2, Nærum, Denmark), was inserted between the speaker and the ear canal at the entrance of the canal to measure the sound pressure level (SPL) inside the sealed volume. To compensate for the frequency-dependent sensitivity of the speaker, the generated sound pressure was corrected to 90 dB SPL for all frequencies in a single iteration. The frequency-dependent response of the microphone was accounted for. Sound-induced vibrations of the footplate were measured from the medial side of the ear using a single-point one-dimensional laser Doppler vibrometer (Polytec, OFV-534 sensor head and OFV-5000 controller, Waldbronn, Germany) that was mounted on a surgical microscope (Carl Zeiss, OPMI Sensera/S7, Jena, Germany). By using the microscope, the beam of the laser was pointed at the centre of the footplate perpendicularly to the footplate surface, and to increase reflection of the laser on the sample, a miniature piece of reflective foil was fixed on the surface of the footplate which was small enough to minimize inertial effects. To monitor how well the sound source was sealed in the ear canal, a second probe tube microphone was positioned right in front of the medial side of the footplate, of which the recorded SPL was compared to the sound pressure detected at the entrance of the ear canal. The resulting stimulation and response signals were retrieved using a custom-made Matlab program (Mathworks, Natick, MA, USA) that communicates with a data acquisition module (National Instruments, USB-6251 BNC, Austin, TX, USA). The sample rate of the input and output signals was set at a frequency of 50 kHz. Each signal was extended for 0.1 s to exclude transient effects in the stimulation and response signals. Finally, the amplitude of each signal was calculated from the Fourier transform of the waveforms at the corresponding stimulus frequencies. This experimental procedure was performed on the heads of both hens and roosters, and repeated for closed and maximally opened beak conditions.

#### 2.1.3. Statistical analysis

To determine whether the observed difference in attenuation between male and female chickens was statistically significant, a statistical analysis was performed in R 2.15.1 (R Core Team, Vienna, Austria). The data to be analysed were the attenuations in each specimen averaged over frequency. A Shapiro–Wilk normality test was performed to verify whether the data were distributed normally (*W* > 0.9). When normality could be confirmed, an *F*-test was executed to compare variances (*p* < 0.05: variances not equal). Normality and equal variance were met for all data. Afterwards, a one-way ANOVA was performed in which the *p*-value was determined to verify whether or not the null hypothesis, i.e. no difference between the sexes, could be rejected. Additionally, the statistical power was determined for a significance level of 0.05 and the given sample size.

### 2.2. Rooster vocalization experiments

To study both the amplitude and frequency characteristics of a rooster's crow in the proximity of its own ears, a miniature audio recorder (Wristband Voice Recorder, J&R Electronics, Hong Kong, China) was used to record vocalizations of an adult domestic rooster. The sensitivity of the device was attenuated so that SPLs up to 140 dB SPL could be measured without saturation. The rooster was 5 years old and had a body weight of 3.2 kg. The audio recorder was incorporated in a wristband that was suspended loosely around the neck of the animal right under the head, with the microphone positioned near the ear. The wristband was put on in the evening so the animal was accustomed to it before crowing at dawn. The audio recorder had a sample rate of 16 kHz and a bit depth of 16 bit, and was calibrated using a pre-calibrated microphone (Brüel & Kjær, Probe Microphone Type 4182, Nærum, Denmark) by measuring the SPL of stepwise pure-tone sound signals generated by a loudspeaker. In this procedure, the audio recorder and microphone were positioned at the same location at a distance of 20 cm from the loudspeaker to measure the SPL under free-field conditions. As a result, the frequency-dependent calibration curve of the audio recorder could be obtained by comparing the magnitudes of both signals. Subsequently, the total SPL of the vocalization signal as a function of time was calculated by means of a standard Fourier analysis procedure.

## 3. Results

### 3.1. Middle ear vibration experiments

#### 3.1.1. Female specimens

**Figure 2.**
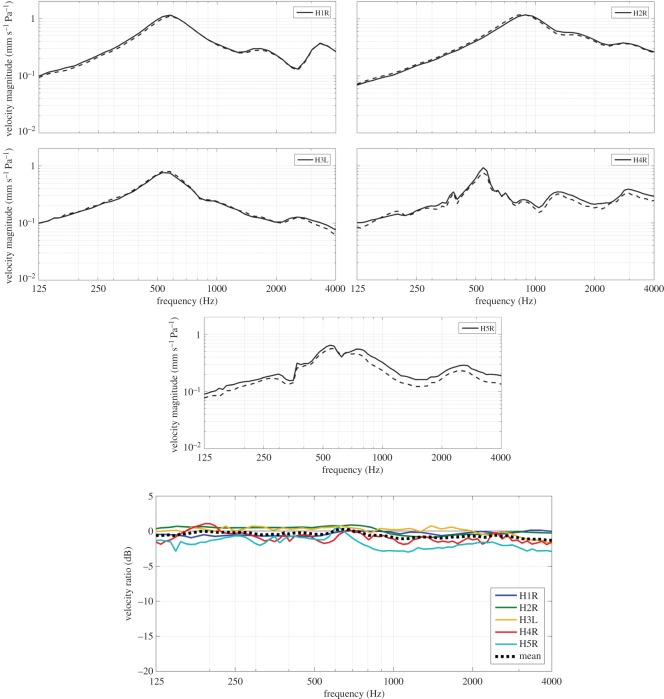
Footplate vibration in hens in response to sound pressures applied at the entrance of the ear canal. Five top panels: magnitude of the vibration velocity normalized to incident pressure (mm s^−1^ Pa^−1^) as a function of frequency, measured with a closed beak (solid lines) and maximally opened beak (dashed lines) for each sample individually. Bottom panel: ratio of the velocity magnitude (dB) of opened to closed beak for all specimens. The black dashed line represents the mean velocity ratio of all samples.

The results of the LDV experiments that were performed on the heads of five hens (H1R, H2R, H3L, H4R and H5R; H = hen; L = left ear and R = right ear) are shown in figure 2. The five top panels in figure 2 show the magnitude of the vibration velocity of the footplate in response to sound pressures introduced at the entrance of the ear canal as a function of frequency. Solid lines represent the measurements performed on a head with a closed beak, while the measurements with a maximally opened beak are depicted by dashed lines. Maximal beak opening in hens corresponds to a lower jaw depression of 34.1° and an upper jaw elevation of 12.7° [7]. When inspecting the results, we observe that each sample contains a peak in the velocity magnitude around a frequency of 0.55 kHz for both a closed and opened beak. There is one exception to this rule, namely sample H2R, which exhibits a peak resonance near 0.9 kHz. The maximum peak amplitude is around 1 mm s^-1^ Pa^-1^. In some specimens, a minor second peak is found between 1 and 2 kHz and a third one above 2 kHz. To quantify the loss in footplate vibration amplitude of the opened beak configuration with respect to the closed beak configuration, the ratio of the velocity magnitude of both conditions was calculated for each specimen, as is shown in the bottom panel of figure 2. The black dashed line in this figure represents the mean velocity ratio of all samples. Ratios are shown in dB, which allows easy interpretation in terms of hearing loss. First of all, the figure shows that the difference in velocity magnitude between a closed and opened beak is small for each sample. Only for specimen H5R, we note that a small decrease in vibration response is seen for the results with an opened beak, but the observed drop in this sample remains smaller than 3 dB over all frequencies. All other samples exhibit a negligible loss of vibration response. The mean velocity ratio over all samples of opened to closed beak is as small as 1 dB or less for all frequencies, with an average value of around 0.5 dB over frequency.

#### 3.1.2. Male specimens

**Figure 3.**
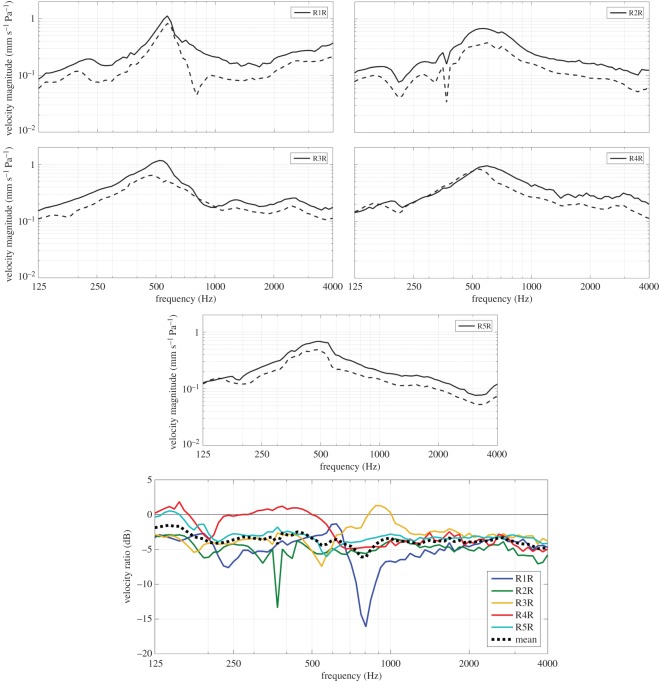
Footplate vibration in roosters in response to sound pressures applied at the entrance of the ear canal. Five top panels: magnitude of the vibration velocity normalized to incident pressure (mm s^−1^ Pa^−1^) as a function of frequency, measured with a closed beak (solid lines) and maximally opened beak (dashed lines) for each sample individually. Bottom panel: ratio of the velocity magnitude (dB) of opened to closed beak for all specimens. The black dashed line represents the mean velocity ratio of all samples.

The results of the same experiments performed on the heads of five roosters (R1R–R5R; first R = rooster; second R = right ear) are shown in figure 3. In roosters, maximal beak opening corresponds to a lower jaw depression of 32.7° and an upper jaw elevation of 18.5° [7]. As in hens, a peak resonance is observed around approximately 0.55 kHz for all specimens in the top panels of figure 3. In some samples, a second resonance is prominent above 2 kHz. In contrast to hens, however, there is a drop in the velocity magnitude of the opened to closed beak measurements in roosters. When comparing the velocity ratios in the ME of roosters in the bottom panel of figure 3, we detect some variability across the samples below the first resonance, with sample R4R showing no loss in vibration and other samples such as R1R and R2R displaying a decrease of 5 dB or more. For frequencies above the resonance, the velocity ratio is more consistent. Specimens R1R and R2R, however, contain a sudden drop in vibration response at isolated frequencies of 0.37 and 0.85 kHz, respectively. This behaviour is seen in both the velocity magnitude and the velocity ratio of the involved specimens. The mean velocity ratio over all sample ranges between 1.5 and 6 dB, with an average value of 3.5 dB over frequency.

**Figure 4.**
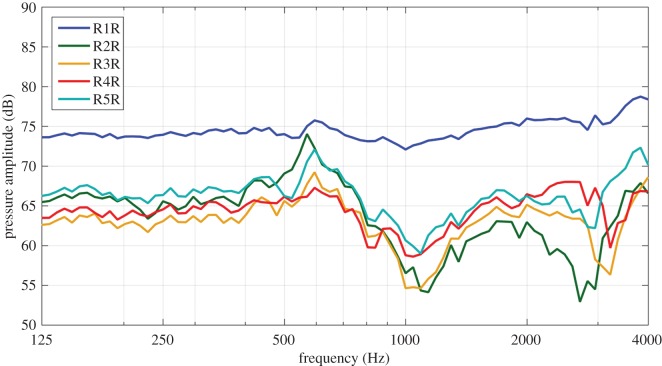
Level of sound stimulus at the footplate in roosters when 90 dB SPL stimulation occurred in the ear canal. This secondary stimulus was at least 15 and generally 25 dB smaller than the ear canal stimulus.

The acoustic cross-talk over the ear that was monitored during the vibration experiments on roosters is shown in figure 4 for the measurements with a closed beak. As can be seen from the figure, the sound pressure reaching the footplate caused by acoustic cross-talk was at least 15 dB SPL smaller than the sound input applied directly at the entrance of the ear canal. In all samples, except for R1R, the SPL was 25 dB smaller than the sound pressure at the ear canal for most frequencies. Near 0.6 and 4 kHz, the acoustic cross-talk was highest in most samples, while it was smallest around 1 kHz and 3 kHz. Results of the acoustic cross-talk with an opened beak were the same as with a closed beak. A similar result was obtained for the experiments on hens.

#### 3.1.3. Statistical analysis

The one-way ANOVA results in a *p*-value of 0.0015225, which is within the region of *p* ≤ 0.05 for which the null hypothesis is commonly rejected. The coefficient of determination *R*^2^ = 0.7018, and the statistical power of the test is equal to 0.9836.

### 3.2. Rooster vocalization experiments

**Figure 5.**
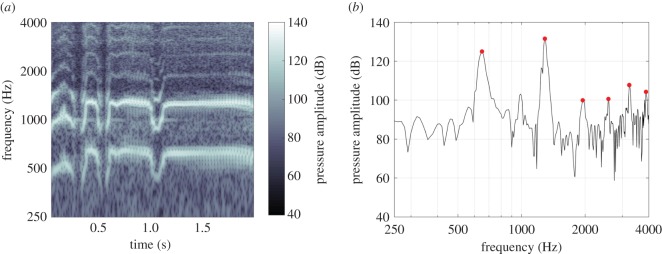
Spectral decomposition of the rooster's crow. (*a*) Calibrated spectrogram of the sound pressure signal (dB SPL re 20 µPa) as a function of time and frequency. (*b*) Frequency spectrum of the time fragment for which the total SPL was maximal, occurring at approximately 0.4 s in the crowing cycle. Red dots annotate the location of the amplitude peaks that are used to calculate the total SPL.

The rooster used for the vocalization experiments was recorded during multiple vocalization cycles. The spectrogram of such a cycle is shown in figure 5*a*, after it was corrected for the calibration curve of the audio recorder. The shade in the plot represents the magnitude of the sound pressure expressed in dB SPL (re 20 µPa) as a function of frequency and time. The spectrogram is shown for frequencies between 0.25 and 4 kHz as this range includes the audible range of chickens.

First of all, we observe multiple peaks arising in the magnitude of the spectrogram: each peak component is a multiple or harmonic of the fundamental frequency, also called the first harmonic or pitch. The fundamental frequency fluctuates between 0.4 and 0.65 kHz in the beginning and middle of the cycle, and reaches a stable value in the second half around 0.6 kHz. The most prominent peaks in the spectrogram are the first and second harmonic, thus being the primary contributors to the total SPL of the vocalization signal.

From the spectrogram in figure 5*a*, the total SPL of the vocalization could be calculated as a function of time. In figure 5*b*, the frequency spectrum is shown of the time fragment in the signal for which the total SPL is maximal, i.e. after around 0.4 s. In figure 5*b*, we can discriminate the six different harmonics whose sinusoidal contributions—each with correct amplitude and phase—are summed to compute the total SPL as the root mean square of this signal. As mentioned in the previous paragraph, the first and second harmonics are the largest in magnitude. With peak frequencies of 0.65 and 1.3 kHz and peak amplitudes of 125 and 131.5 dB SPL, respectively, they are the primary contributors to the total SPL of the vocalization signal at maximal sound intensity.

**Figure 6.**
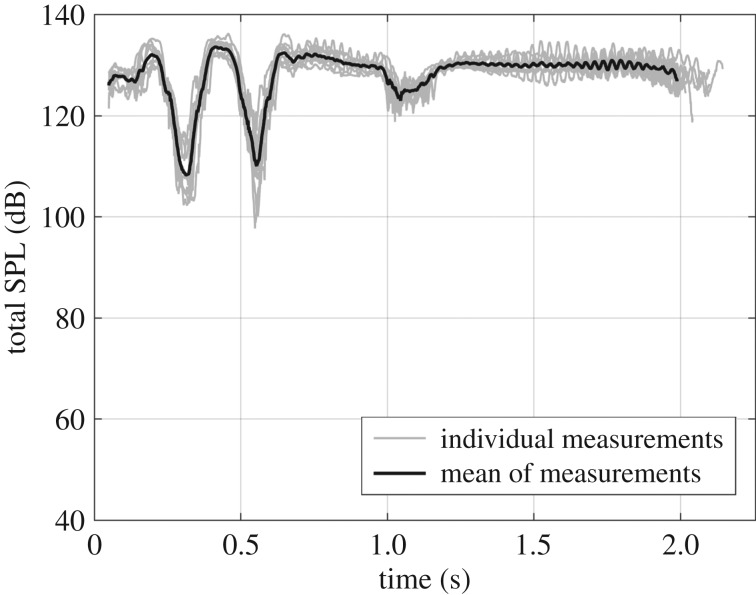
Total sound pressure amplitude (dB SPL re 20 µPa) of all vocalizations produced by the rooster as a function of time.

The total SPL of all vocalizations produced by the rooster is shown in figure 6 as a function of time. The curves show that the vocalization signal is very reproducible: each cycle contains two distinctive SPL minima after 0.3 and 0.55 s, and one moderate minimum after 1.1 s. On average, the rooster produced maximal pressures amplitudes of 133.5 dB SPL close to the ear of the animal after 0.4 s in the crowing cycle. Individual cycles even reached values of 136 dB SPL.

## 4. Discussion

### 4.1 Middle ear vibration experiments

#### 4.1.1 Experimental approach

The *ex vivo* ME vibration experiments allowed us to rule out stapedius muscle activity during measurement, as the current study is concerned with purely mechanical processes in the ear. However, it is well known that after death, the structure of soft tissue changes, which can affect the mechanical properties of parts of the system. The presented experiments are impossible to perform *in vivo,* so we are unable to compare the *ex vivo* measurements to *in vivo* results. However, studies of the ME input admittance in humans (e.g. [26]) have shown that changes after death are generally small if the preparations are kept moist and cool, which suggests that the chosen post-mortem approach is justified.

In order not to influence the natural shape of the ear canal, or the possible change of shape after beak opening, ME vibrations were not measured on the lateral surface of the tympanic membrane but on the medial surface of the footplate. To reach the medial footplate surface, the skull was opened caudally and the inner ear was opened and drained, which also guaranteed that no additional quasi-static pressures were acting on the tympanic membrane that could alter ME response. By sealing the sound source in the ear canal, the acoustic cross-talk described by the difference in sound pressure between near the footplate and the closed ear canal is at least −25 dB. In sample R1R, however, the SPL at the footplate was only 15 dB lower than at the ear canal, which could be caused by a leaky sealing of the speaker. Even a cross-talk of as little as −15 dB should produce an increase in stapes motion of less than 0.1 dB. Moreover, the resulting effect on the velocity ratio of opened to closed beak is non-existent, as the acoustic cross-talk was the same with a closed and opened beak, and because the bird ME was shown to behave linearly up to stimulus levels of 120 dB SPL [16].

Opening and draining the inner ear alters the vibration response of the footplate. For instance, the resonance frequency in the vibration response of the footplate is shifted to higher frequencies due to the presence of the inner ear, as has been reported on ostrich ears [23]. In the current paper, we are investigating the attenuation of an opened with respect to closed beak due to mechanical effects on the ear. Changes in the shape of the canal may reduce the sound pressure reaching the membrane, although our experimental set-up did not allow us to establish to what extent this effectively occurred. When in a linear system the input sound pressure is lowered, the output (in this case the footplate velocity) will be lowered to the same degree. Hence, removing the inner-ear impedance will not influence this part of the attenuation effect. It has been demonstrated that ME nonlinearities exist (e.g. [27]), but the effect is very small. Different from what is found in mammals, motions of the basilar membrane in the bird inner ear do not show any nonlinear behaviour [28], which suggests that the inner-ear load is also linear. Therefore, it is reasonable to assume that the system behaves linearly for the sound pressures encountered during vocalization.

For the attenuation caused by mechanical changes in the tympanic membrane and the ME, the situation is more complicated. Even when the inner ear behaves linearly, the transfer of motion between the input (the tympanic membrane) and the output (the footplate) depends on the acoustic impedance of the ME and the inner ear, and on how the ME impedance changes due to the beak opening. It might be that the columella and extracolumella show more bending when coupled to a stiff inner ear than when acting upon an unloaded footplate. In such a case, the reduction of columellar movement due to beak opening may be different in the case of an intact inner ear than in the case of a removed inner ear. It has been shown, however, that the avian inner-ear impedance, as measured in ostrich, is a factor of 10 to 100 smaller than the inner-ear impedance measured in mammals [23]. In other words, the bird inner ear is a rather compliant structure. Nevertheless, it should be noted that the attenuation of footplate motion caused by beak opening in the absence of the inner ear is not necessarily identical to the attenuation with the inner ear intact.

To measure the effect of beak opening, the beak was opened maximally during measurement. In certain birds it has been shown that louder sounds are produced with larger gaping (e.g. [29,30]), and that larger gapes are related to the production of higher frequencies (e.g. [31–33]). It is not certain that these findings also apply to roosters and, if so, whether they are associated with maximal beak opening during highest sound production.

In the presented experiments, sound pressures were introduced at the entrance of the ear canal. Yet, there remains a possibility that vocally evoked sounds are transmitted by the pharyngotympanic tubes to the medial tympanic membrane surfaces. However, the pharyngotympanic tubes of chickens are closed most of the time and only open occasionally when swallowing [6]. Also in humans the tubes are normally closed and only occasionally open to pass gas between the ME cavity and the nasal cavity (e.g. [34,35]). In humans, a clinical condition exists in which the Eustachian tubes are permanently open. People with this condition often hear their own voice very loudly due to internal sound transmission. In healthy people with a closed Eustachian tube, we can estimate the SPL reaching the medial tympanic membrane surface relative to the lateral membrane surface due to self-generated sounds. Kawase *et al*. [36] found that the SPL in healthy subjects was around 20 dB higher in the nasal cavity than in the ear canal. Measurements in patients with open Eustachian tube have shown an increase in the auditory threshold of 40 dB after treatment of the tube (i.e. closing the tube) in response to sound pressures introduced in the nasal cavity [37,38]. When we assume that the SPL behind the tympanic membrane is as large as the SPL in the nasal cavity in people with an *open* Eustachian tube, then the SPL in subjects with *closed* tubes will be around 20 dB higher in front of the tympanic membrane than behind the membrane. In people with an open Eustachian tube, however, the actual sound pressure in the ME will be lower than the SPL in the nasal cavity due to attenuation of internal transmission. Therefore, the difference of 20 dB for closed tubes is merely a lower bound, so the effect of internal transmission remains limited.

#### 4.1.2. Experimental results

In roosters, the decrease in vibration response of the footplate was shown to be significantly larger than the decrease observed in hens, i.e. 3.5 dB in roosters against 0.5 dB in hens on average. The possible source of the observed decrease in vibration response is a change in tension of the tympanic membrane. In chickens, the skull is prokinetic, which implies that movements of the bill are a combination of lower jaw depression and upper jaw elevation [39]. According to Ewald [10], the skin covering the lower jaw pulls on the skin of the ear canal due to beak opening, which in turn pulls on the tympanic membrane and changes its tension. On the other hand, the quadrate performs rotations that are associated with elevations of the upper jaw [39–41], which could lead to changes in tympanic membrane shape and tension [4]. Claes *et al*. [7] compared craniokinesis of the sexes in the domestic chicken and found a difference in upper jaw elevation, with roosters displaying a greater upper jaw lift than hens. As a result, roosters potentially exhibit larger gaping moderated by the quadrate. The more pronounced upper jaw elevation in roosters goes along with larger rotational angles of the quadrate, potentially leading to greater structural changes to the ear of roosters. Claes *et al*. [7] also observed clear differences in tympanic membrane displacements in both ears of one hen and one rooster. In hens, the shape of the conical tympanic membrane remained unchanged and the conical tip of the membrane did not move when the beak was opened. In roosters a substantial change in position of the conical tip of the tympanic membrane was detected, corresponding to a flattening of the membrane. The observed displacements produced a relaxation of the membrane, which can be a cause of the observed 3.5 dB decrease in vibration velocity.

Another possible mechanism is related to the shape of the ear canal. The canal wall contains an erectile auditory pad—a semicircular worm-like elevation composed of connective tissue and venous spaces, which is better developed in male than in female chickens [2]. This structure was first described by Wurm [42] and von Graaf [43] in capercaillie, arguing that it is responsible for the temporary deafness during sexual excitement by plugging the ear canals. Later, von Békésy [44] observed that the entrance and the cross-sectional area of the rooster's ear canal become visibly smaller while raising the head during vocalization. The ear canal connected to the closed volume of the ME could act as a low-pass filter. Decreasing the diameter of the canal increases its resistance, which lowers the cut-off frequency. Therefore, high-frequency sound components could be attenuated more when the ear canal becomes narrower. However, during the current experiments it could not be observed whether the opening of the ear canal effectively became smaller, due to the acoustical seal made of modelling clay applied around the canal entrance. Therefore, it is likely that the attenuation observed is only related to a mechanical effect on the tympanic membrane.

Despite the significant difference in attenuation between hens and roosters, the attenuation observed in the current study remains limited, providing little effective protection to the inner ears. Therefore, effective protection from self-generated vocalizations in roosters must be more importantly obtained through other mechanisms. Ewald [10] suggested a possible role of beak opening on the static inner-ear pressure, which may result in a decrease of the cochlear response. Bray & Thurlow [11] concluded from their measurements on pigeons that the observed 20 dB drop in cochlear response is a result of a rise in inner-ear pressure alongside a change in tympanic membrane tension. Therefore, the difference between the presented decrease of 3.5 dB and the drop of 20 dB observed by Bray & Thurlow [11] may be partially due to a change in inner-ear pressure. Additionally, it has been shown that stapedius muscle activity in roosters causes attenuations up to 20 dB [8,45,46]. Therefore, the presently described effect is clearly inferior in magnitude. Another potential mechanism is that roosters open their pharyngotympanic tubes while crowing, allowing sound to pass through, which leads to transtympanic pressure loads that destructively interfere with (and thus attenuate) the direct pressure load on the tympanic membrane. The existence of such a mechanism, however, remains speculative at present.

### 4.2. Rooster vocalization experiments

The experiments show that the maximal sound pressure produced by the roosters measured close to the ear of the animal amounts to 133.5 dB when averaged over multiple vocalizations. Individual vocalizations even reach values of 136 dB. Brackenbury [47] recorded rooster vocalization at a distance of one metre from the animal and obtained values of 100 dB, which corresponds to an average sound power that is approximately 27 times larger than the maximal speech power produced during very loud human conversation [48]. Borg and Counter [46] reported an SPL of 130 dB measured at the head of the rooster, which is similar to our presented results. Such high pressure levels are above the commonly considered threshold of pain, which amounts to 120 dB, and is potentially harmful to the inner-ear receptor cells. This result gives suggestive evidence for the need of protective mechanisms in the ear of roosters. As hens do not crow, they are less in need of such a mechanism in response to self-generated vocalizations.

Vocalizations are made up of harmonic series with a fundamental frequency around 0.6 kHz in the second half of the vocalization cycle. The two most prominent peaks in the spectrogram are the first and second harmonic. When relating the results of the vocalization recordings to the ME vibration experiments, it is observed that the first harmonic in the crowing signal at the maximal SPL (figure 5*b*) approaches the first resonance of the footplate response in the rooster ME (top panels in figure 3), which is around 0.55 kHz. The frequency of best hearing in chickens lies around 1.41 kHz [25], which is closer to the second harmonic in the vocalization signal (figure 5*b*). As overly loud sounds associated with vocalization are in the sensitive region of the chicken, some degree of auditory protection is needed. Sound attenuation related to beak opening shows no preferred frequency region, because the velocity loss of the footplate vibrations during beak opening is constant as a function of frequency (bottom panel in figure 3). Stapedius muscle activity, on the other hand, has been shown to mainly influence low frequencies [46], so the effect will be strongest around the first and second harmonic in the vocalization signal.

## 5. Conclusion

Footplate vibration attenuation as a result of beak opening is fairly constant as a function of frequency, with an average vibration loss of 3.5 dB in roosters and 0.5 dB in hens. Although also in roosters the loss is small, the difference between hens and roosters is statistically significant. As observed by Claes *et al*. [7], the dissimilarity between the sexes may be related to a difference in upper jaw elevation, and hence quadrate motion. The interpretation for the origin of the attenuation is the following: the kinetic quadrate causes change in the shape and tension of the tympanic membrane [7], which changes the transmission properties of the ME. Vocalization recordings show maximal sound pressures of 133.5 dB SPL on average, and a frequency content that is in accordance with the hearing range of highest sensitivity in chickens. The obtained values are above the threshold of pain and are potentially harmful to the inner ear receptor cells, thus supporting the idea that a protective mechanism is needed for self-generated vocalizations. The attenuation related to beak opening is limited to about 3.5 dB, which will provide little effective protection to the inner ears. Therefore, effective protection from loud self-generated vocalizations must be obtained by combining the mechanical effect of beak opening with other mechanisms such as stapedius muscle activity.

## Data accessibility

Our data are deposited at Dryad: http://dx.doi.org/10.5061/dryad.fr684 [49].
